# Assessing Urban Policies in a COVID-19 World

**DOI:** 10.3390/ijerph19095322

**Published:** 2022-04-27

**Authors:** Przemysław Śleszyński, Paulina Legutko-Kobus, Mark Rosenberg, Viktoriya Pantyley, Maciej J. Nowak

**Affiliations:** 1 Institute of Geography and Spatial Organization, Polish Academy of Sciences, 00-818 Warsaw, Poland; psleszyn@twarda.pan.pl; 2 Department of Public Policy, Warsaw School of Economics (SGH), 02-554 Warsaw, Poland; plegut@sgh.waw.pl; 3 Health and Development, Queen’s University, Kingston, ON K7L 3N6, Canada; mark.rosenberg@queensu.ca; 4 Department of Social and Economic Geography, Maria Curie-Skłodowska University, 20-031 Lublin, Poland; viktoriya.pantyley@mail.umcs.pl; 5 Real Estate Department, West Pomeranian University of Technology, 70-310 Szczecin, Poland

**Keywords:** urban politics, COVID-19 pandemic, social issues, spatial organization, public authority, future of spatial and urban policy

## Abstract

The aim of this study was to identify how the literature analyzes (identifies, evaluates, forecasts, etc.) the relationship between health issues and urban policy in relation to the COVID-19 pandemic. Four main levels were identified in these cases: (1) direct demands for changes in health care, (2) social issues, (3) spatial organization and (4) redefining the tasks of public authority in the face of identified challenges. The basic working method used in the study assumed a critical analysis of the literature on the subject. The time scope of the search covered articles from January 2020 to the end of August 2021 (thus covering the period of three pandemic waves). Combinations of keywords in the titles were used to search for articles. The health perspective pointed to the need for urban policies to develop a balance between health and economic costs and for coordination between different professionals/areas. A prerequisite for such a balance in cities is the carrying out of social and spatial analyses. These should illustrate the diversity of the social situations in individual cities (and more broadly in urban areas, including, sometimes, large suburbs) and the diversity’s relationship (both in terms of causes and consequences) to the severity of pandemics and other health threats.

## 1. Background

The COVID-19 pandemic has introduced a need to redefine many urban policies. Sharifi and Khavarian-Garmsir [1] identified several key levels of discussion in this context: urban environmental quality, the socio-economic sphere, governance, transport and urban planning. There is no doubt that a key emerging pandemic-related issue is also the clarification of the relationship between urban policy and health care. Such a demand in the general sphere has been formulated in numerous publications [2]. For example, Nieuwenhuijsen [3] pointed out a need to link health care issues with spatial planning. There is no doubt that clarifying the role of health care in select urban policies is becoming a serious challenge. The relationship between health issues and urban policies prior the COVID-19 pandemic was a subject of analysis both in terms of select aspects of such an analysis [4,5] and in terms of select regions in the world [6,7]. The issue of the direct (pandemic-induced) relationship between health care and pandemics has already been partially addressed in the literature. Mostly, however, it has been isolated in the contexts of other issues. Sharifi et al. [8] linked the context of urban policy and health with climate change adaptation efforts. However, they framed the issue not so much from the perspective of the relationship between health care and urban policy itself (in relation to pandemics) but from the perspective of finding common ground between health care and climate change responses. A similar direction was taken by Sheehan et al. [9], recognizing that health adaptation focuses on mapping risks and managing climate-related public-health capacity building. The link between social and health dimensions in cities was noted by Alizadeh and Sharifi [10] and Khalili et al. [11], among others. The social and environmental thread was also linked to the challenges of climate change adaptation [12,13]. What is missing from the ongoing discussion is a comprehensive analysis of the very relationship between urban policy and health. The purpose of this study was to fill the research gap into this context and determine how the literature analyzes (identifies, evaluates, forecasts, etc.) the relationship between health issues and urban policy. Four main levels were identified (the selection of which is justified in detail in the methodological section): (1) direct demands for changes in health care, (2) social issues, (3) spatial organization and (4) redefining the tasks of public authority in the face of indicated challenges. Already at this point, it should be pointed out that the article deals with the linkages between health care issues and urban policy, and the most relevant issue is direct demands for changes in health care (point 1). However, the authors believe that it is necessary to verify to what extent the health care context appears in the analyses of the other three topics. Only this approach provides a complete picture. It was assumed that a combined analysis of the indicated issues would definitely better enable scholars to identify the most frequent and important trends contained in the literature. Thus, an attempt was made to synthesize indicated recommendations, indicating both well-established postulates as well as those requiring supplementation. A discussion on relationship between health issues and urban policies required at least one more research perspective, taking into consideration area-based aspects of general urban policies and urban health policies in particular [14]. This was due to increasing socio-economic and health inequalities among urban dwellers associated with the COVID-19 pandemic [15,16,17,18]. Area-based policies are directed at decreasing health inequalities and improving the life quality of residents in socio-economically disadvantaged areas of cities. Some findings from Europe (e.g., urban areas of Andalusia in Spain) showed that a combination of area-based policies and urban interventions could generate a significant decrease in preventable and all-cause mortality among residents in SED areas in comparison with control areas (…).

An important caveat is that the focus of this study was on linking isolated urban policy issues to health. Thus, an issue that falls within health policy was extensively examined in the context of the COVID-19 pandemic [19]. In contrast, the discussion of pandemic management is something different from urban policy [20]. The authors of this paper argue that the discussion of integrating health aspects into urban policies, despite individual systemic differences across countries [21], is a largely universal discussion. This is due to several reasons. First, the organization of urban space is universal in the sense that for the vast majority of cities, regardless of their sizes, the same functional zones can be delineated (multifunctional areas, service downtowns, residential areas, workplaces, leisure zones, etc.). This affects the daily mobility of residents. Hence, second, the ways of managing such areas must be similar. In this case, the burden of management is borne by local communities and local governments, although the ways of selecting such communities and governments as well as detailed legal solutions vary. Third, technological convergence is taking place around the world, and the introduction of specific solutions, e.g., infrastructural ones, depends on only levels of development and available financial resources. Fourth, and finally, in the settlement system, cities act as population service centers for wider regions, including their surroundings, such as suburban zones and rural hinterlands. This servicing role includes the location of health care institutions—that is, the larger the city, the more specialized these institutions tend to become and the more they serve a larger territory.

As indicated above, an overview of urban policy issues linked to the medical and pandemic dimensions was provided by Sharifi and Khavarian-Garmsir [1], strongly noting the health sphere in conjunction with social and environmental issues. This study was primarily an attempt to continue and elaborate on the considerations indicated. There is no doubt that health issues (understood in a broad sense) should be linked to adaptation to climate change and shaping the resilience of cities in this context. However, in order to make this possible, it is necessary to identify the main areas in relation to which such a discussion should be held.

A redefinition of the role of public authorities should be considered as very important from the perspective of the subject under consideration. Even before the pandemic, numerous publications drew attention to a need for action in this area, also in relation to health care [22,23]. This was particularly noticeable in urban governance. It is in cities that numerous authors rightly see a dynamic system that is very difficult to sort out comprehensively [24]. This is why the discussion on including diverse stakeholders in urban management was so important [25]. Pandemic contexts (and the increasingly visible challenges of responding to climate change) deepen these needs [26]. A growing number of perspectives are emerging that are important to consider in urban governance. There is no doubt that one of the most important of these perspectives is health. So far, in many countries—if due only to underdeveloped concepts of integrated development planning [27]—the wider inclusion of this perspective in urban management has been a major problem. The pandemic reflection should definitely change this.

The methodological section carefully justifies the selection of issues and the search for publications. Then, in the overview section, the key theses and trends in each of the four issues are presented (distinguishing, on the basis of the collected publications and their theses, minor issues, if possible). The discussion section then comments on the trends and, where possible, suggests further directions for scientific discussion. In the last part, the key conclusions of the article are given.

## 2. Materials and Methods

The basic method used in the study was a critical analysis of the literature on health care issues and urban policy. In order to achieve the aim of the study, the authors adopted a multi-stage approach. It is worth noting that even before the pandemic, the literature on the subject recognized and postulated the need for a broader link between health care and urban spatial policies [28]. This direction became even more relevant after the pandemic began [29].

On the basis of a preliminary review of the literature, including, in particular, the multi-criteria analyses carried out so far related to the development of the pandemic and the author’s own research, 4 basic thematic issues were formulated, which constitute a kind of ‘axes’ for analysis: (1) direct demands for changes in health care, (2) social issues, (3) spatial organization and (4) redefining the tasks of public authority in the face of indicated challenges. Specific issues were also identified for each main issue (presented in Table 1, summarizing the analysis).

The next research step was to search the database for articles. The authors decided to use the software Publish or Perish [30] based on the Google Scholar search engine. This is a way to significantly expand the potential article database in comparison to, for example, Scopus. The time scope of the search covered articles from January 2020 to the end of August 2021 (so it covered a period of 3 pandemic waves). Combinations of keywords in titles were used to search for articles. The keywords were selected based on the authors’ expert analyses and included the following associations: one group of words was COVID, SARS-CoV-2, pandemic, epidemic and coronavirus with words from a second group, which were cities, city, town, urban, spatial policy, public health, social exclusion and social inclusion health education. The search was conducted in such a way that two groups of words were combined in each variant. This search yielded a total of 10,310 indications, most of which (as many as 5743) appeared when the first word of the analysis was COVID (Table 1).

Publications on European countries were selected from this database and officially categorized as scientific as they were selected. This stage of the analysis proved to be the most time consuming as many publications either marginally or not at all addressed the issues of interest in this article; despite the wordings in the titles suggesting such an approach, many publications were presentations of the results of clinical studies analyzing the dynamics of incidences and vaccinations in specific cities. These topics were not the focuses of our analysis.

The process of selecting articles in this way resulted in 240 articles for analysis. They referred to individual main and detailed issues (Table 2; it should be noted that some articles concerned more than one issue, so the data presented in the table do not add up to 240). The articles that were analyzed could be divided into several groups. These were:

- Individual case studies of specific cities (that most often referred to London, Madrid, Paris and Barcelona).
- Case studies of European cities, but in the context of other countries (most often China and USA).
- Comparative case studies covering different categories of cities in one country (e.g., Germany’s small and medium-sized cities).
- Comparative case study covering cities from several countries (e.g., Sweden, Norway and France).
- Comparative studies covering one analyzed topic in different cities (e.g., bicycle transport or participation).
- Comparative analyses covering different cities from around the world (usually selected on the basis of the course of the pandemic).
- Comparative analyses including urban policy responses to COVID-19 in different countries.
- Analyses of trends in and evolutions of changes in urban policies (usually theoretical, based on the literature).

Articles categorized under each topic area formed the basis of the literature review. References are made to the broader literature in the introductory section and in the discussion and conclusions.

## 3. Results (Literature Review)

### 3.1. Direct Changes in Health Care Systems

The issues of direct changes in health care systems during the COVID-19 pandemic were addressed in 41 scientific publications (these were mainly scientific articles as well as analytical reports of organizations dealing with health care in a broad sense). Within health issues, the most frequent were publications on the reorganization of health care systems, the promotion of healthy lifestyles to urban residents and However, publications on health service resources and operations as well as on the financing of health services in cities were relatively less frequent.

In research articles addressing the reorganization of urban health services, a key concept was that of disaster risk management and pandemic-resilient urban strategies related to health. Afrin et al. [31] pointed out the necessity of introducing disaster risk management into urban health systems to help develop pandemic-resilient urban strategies, taking into account such management’s following phases: response, mitigation and preparedness phases. Population health, considered through the lens of housing–city–public spaces interconnectedness, plays a key role in disaster risk management. An article by Rice [32] highlighted the role of urban design in determining the health of urban residents, but this relationship was often ‘unclear and undervalued’. Afrin et al. [31] provided short- and long-term recommendations for pandemic-resilience urban planning and design. In this context, it is particularly important to learn about the various aspects of resilient urban strategies (such as infrastructure, environmental, political, socio-cultural and governance factors) to help better understand health and disaster-related risks in a pandemic. Such strategies may be helpful should a pandemic occur in the future. Examples of strategies are given in Bell [33], for Sweden and Norway, and in Moatti [34], for France.

Ding et al. [35] emphasized that in European countries with higher health-sector capacities, the course of the epidemic was more severe. It was pointed out that the capacity of the health system in Western Europe is higher than in Northern, Southern and Eastern Europe. This is because of the larger area, larger populations and better socio-economic conditions in western European countries in comparison with other parts of Europe. On the contrary, with respect to health care system capacity, the general development of the public health care sectors of Western Europe for the past ten years was not balanced and stable, while it remained stable and balanced at a high level for Northern Europe.

Megahed and Ghoneim [36] considered urban population health from the perspective of the pillars of sustainable development. In their opinion, it seems necessary to add population health as a fourth pillar to the general definition of sustainable development. This was also mentioned in work by Pantyley in relation to Ukraine [37].

Issues of health care reorganization and the evaluation of health-policy effectiveness in the face of the COVID-19 pandemic were included in recent WHO reports. One thematic WHO paper [38] provided a methodology for calculating the PHSM (Public Health and Social Measures) Severity Index, constructed as ‘capturing, coding, visualizing and analyzing PHSH responses to COVID-19’ in WHO European-region countries. This index can be helpful in assessing the effectiveness of health policies at state and regional levels. In turn, another WHO strategy document [39] indicated an urgent need for health service reorganization, decentralization and cooperation with local communities. Szmytkowska [40] signaled there is a problem of a lack of or insufficient availability of data on monitoring the health statuses of urban residents.

Pinto et al. [41] highlighted issues related to monitoring the health statuses of urban residents from a historical perspective. According to the authors, emphases should be on urban health security requirements as well as on design solutions to restore lost synergies between communities and places. Falanga [42] wrote a paper devoted to monitoring the health statuses of urban residents in the face of a contemporary pandemic. The paper included examples of short-term participatory actions in countries, such as France, the UK and Finland, aimed at giving practical support in different domains of social policy through the possibilities of the digital society.

Megahed and Ghoneim [36] introduced the concept of ‘anti-virus-built environment’, assuming that many architectural and urban planning solutions can increase the protection of urban spaces and avoid overcrowding. This work emphasized the importance of distributing smaller service units, such as health facilities, schools and services, across a larger portion of the urban fabric and strengthening local centers during pandemic periods (also see Alter [43] and Wainwright [44]). As Smith and Quale’s [45] research indicated, a modular construction strategy, increasingly popular before COVID-19, is effective in the face of pandemics or natural disasters in creating cheaper- and faster-constructed buildings. Modular construction elements can help buildings adapt or expand their spaces for treatments and quarantines [46]. Hassan and Megahed [47] developed concrete architectural solutions—nine models—to maintain the good health of urban residents during the COVID-19 pandemic. Parmet [48] emphasized the role of safe urban public spaces in maintaining the good health of residents during the pandemic.

Sharifi et al. [49] analyzed the issues of the health-care services sector as well as better pandemic management taking advantage of the ability to track, diagnose, supervise and treat patients in cities through smart city solutions (such as artificial intelligence, the IoT and drones). Esposito [50] highlighted the existence of a great need for ‘data-driven solutions for infection tracing and forecasting epidemic trends’, which are essential to achieve sustainable and socially resilient cities. Such solutions include DPTT (Digital Proximity Tracing Technology) and DDEIS (Data-Driven Epidemic Intelligence Strategies). Shahbazian [51] focused on practical solutions toward designing cities to ensure the health of their inhabitants. Such solutions include: the ‘expansion of cycling infrastructure, expansion of green spaces and public open space, lack of focus on public facilities in one place, housing design and home design strategies in the face of COVID-19, management of resource consumption, especially water resources’ and ‘health and waste management’. Sharifi et al. [49] suggested that the further development of smart city initiatives may confer unprecedented opportunities toward strengthening urban resilience to pandemic challenges and similar events that may arise in the future. Barriers to such opportunities will be socio-economic and institutional constraints, especially for local authorities as indicated, for example, by the research of Jambrović [52] on cities in Croatia.

A study by Luzi et al. [53] pointed out the necessity of an increasing role of the prevention of other chronic diseases in the face of contemporary pandemic challenges. The paper analyzed the potential relationships between the prevalences of diabetes, obesity and incidences of COVID-19 in the Milano Metropolitan Area and pointed out the necessity of modifying the management of the chronic diseases of the city’s inhabitants with the continuous surveillance of sick people through the implementation of telemedicine technologies. Related issues were addressed in a study by Pelizza and Pupo [54] on the future directions of the public mental health system in Italy (which were telemedicine, online treatments and remote interventions). The article pointed out a need to increase the effectiveness of public health policies toward the ‘strengthening of in-home treatments instead of hospital-centered care’. McDougall et al. [55] signaled that there is an increasing role of other challenges to maintaining good mental health, such as a lack of or poor physical activity among urban residents and inequalities, in where people live. They considered cities in terms of not only ‘places for ill-health prevention but also places of health promotion’.

Acharya et al. [56] identified the roles of health-service resources and activities as well as the role of health-service funding in controlling the course of the COVID-19 pandemic in the UK. Namely, the course of the pandemic was controlled faster in more dense and wealthier areas of the UK. However, there was no correlation between health expenditure from local budgets and the speed of the control of the pandemic in these areas.

Giraud et al. [57] highlighted the roles of health resources and activities in three European countries: France, Germany and Italy. The general trend with respect to the social policies of the three countries in relation to health moved toward marketization and increasing the role of individuals’ responsibility for their own health. In terms of the concept of solidarity, the authors pointed out its strengths and weaknesses with respect to each of the countries analyzed. The pandemic showed ‘deficits in the functioning of government and the health system, but it is yet difficult to predict whether this crisis will contribute to promoting a move away from neo-liberal concepts in favor of recognizing public health and scientific research as collective goods in which governments should invest substantial structural resources’. It was concluded that the management of the COVID-19 pandemic requires coordinating the efforts of public, private and NGO structures at different territorial levels.

At a different geographic scale, Cave at al. [58] analyzed measures of socio-health policy as a result of the severity of the COVID-19 pandemic in four global cities: Sydney, Milan, Seoul and London. The authors provided recommendations toward structural determinants and achieving equity in response to COVID-19 challenges.

### 3.2. Social Issues

Social issues were addressed in 58 publications—these were mainly scientific articles and applied reports. The analyzed publications focused mainly on the following issues: social determinants of health (34 publications) and the reduction or elimination of health inequalities (20 articles). Other topics were addressed less frequently: special measures for the most vulnerable social groups of populations (17 publications); special measures for the most vulnerable demographic groups of populations (10 publications); and urban health education (9 publications). Many of the articles only marginally (signally) addressed social issues related to the pandemic. The COVID-19 pandemic exposed several systemic failures and injustices in the way cities are planned and designed around the world [59]. Researchers pointed out that the COVID-19 pandemic ended urban life as it existed; it also put existing inequalities and poverty under the microscope, and the effects of the pandemic were unevenly distributed across populations, often exacerbating pre-existing inequalities [15].

Only one article in the detailed analysis was considered a comprehensive approach (covering all the social issues considered in detail). In it, Hoernke [60] addressed issues of social justice by pointing out that the pandemic exposed the social inequalities woven into the functioning of modern cities around the world. The author emphasized that exposures to COVID-19 and the effects of the pandemic (including death) are correlated with socio-economic disadvantages and are particularly acute in rapidly urbanizing cities, where there are also the greatest social inequalities, including health and hygiene opportunities (e.g., lack of running water). He saw digitization as an opportunity to reduce social inequalities, particularly with respect to access to knowledge and health education, pointing to digital inequalities and exclusions in this area as well. Wilkinson [61] provided an analysis of social inequalities associated with living in informal urban settlements; she noted that cities are often segregated along wealth and social lines (including race). Images of ‘slums’ depicted such regions as chaotic, dirty and disease ridden and as social, environmental and developmental threats to the rest of cities. She also pointed to another challenge related to social inequalities—people living day to day without stable employment cannot afford to be sick because then they will lose their basis of existence. A detailed analysis of social inequalities in COVID-19 incidences, stratified by age, sex, geographic area, and income in Barcelona during the first two waves of the pandemic, was presented by Marí-Dell’Olmo et al. [17]. As indicated in the study, incidences of COVID-19 were higher in some poor neighborhoods of the city; moreover, inequalities in COVID-19 incidences in the urban area were demonstrated.

Wray et al. [62] emphasized that the COVID-19 pandemic is a crisis of environmental justice and equity in the public sphere. Studies in Spain indicated that the number of deaths caused by COVID-19 is positively related to GDP per capita and inversely related to expenditures on hospital and specialized services and teaching and health resources in the budgets of autonomous communities over the last nine years [63].

The reviewed literature indicated not only social inequalities related to socio-economic factors but also disability inequalities. As the studies indicated, during pandemics and other emergencies (e.g., climate-change-related emergencies), people with disabilities may be four times more likely to be at risk of life-threatening injuries or death due primarily to failures to consider their needs in urban planning and emergency health-system management [64]. As reported by Milner et al. [65], the negative effects of COVID-19 are unevenly distributed across societies, with the impacts being much greater among socio-economically disadvantaged and vulnerable populations. USA research suggested that increased exposures to COVID-19 risk factors are rooted in racial inequities—Black and Latinx people are more likely to be exposed to COVID-19 as they are more likely to have jobs in underpaid ‘essential industries’, which require in-person or face-to-face contact, and because of gender and sexual minority inequities [66].

Fransen et al. [67] pointed to a need to build community resilience and possible pathways to reach the most vulnerable communities that are difficult to reach. They identified four possible pathways: (1) informal grassroots community initiatives; (2) formal community initiatives growing out of existing community initiatives; (3) initiatives by external actors, often NGOs, universities or governments and (4) networks of organizations that collectively initiate actions in response to COVID-19. As the authors pointed out, these pathways allow for a variety of initiatives of varying scale and complexity, but they all face barriers related to funding, weak networks and limited collaborations.

The pandemic and its effects also contributed to a discussion on the concept of more friendly and socially just cities. Córdoba-Hernández [68], using the example of Madrid, pointed out how social inequalities are generated by the places where we live and how challenging it is to redesign spaces in the direction of the ‘15-min city’ originally proposed in the ‘pre-COVID’ reality [69], which is also a more socially inclusive city. The idea of age-friendly cities, including aspects such as outdoor spaces and buildings; transportation; housing; social participation; respect and social inclusion; civic participation and employment; communication and information and community support and health services, requires rethinking and research on how well this concept works in a pandemic [70]. The literature also called for a re-examination of the smart city concept and the role that technology can play in shaping public health and urban functioning [71].

Afrin et al. [31] drew attention not only to a necessity for a new approach to the design of public spaces providing the possibility of maintaining social distance and increasing pedestrian traffic but also to a new approach to the design of housing in such a way that it provides the possibility of adapting the functioning of entire families to changing conditions during pandemics and similar catastrophic phenomena. A dwelling should become not only a place to live but also a place to work and study. In this context, it is worth referring to research conducted in three cities in Sweden (Stockholm, Uppsala and Gothenburg), which clearly indicated that the pandemic reinforces existing social inequalities in cities, and in urban areas characterized by poverty and overcrowding, green and publicly accessible areas are increasingly important [72]. Research in the USA found that access to urban green space is unequal, with several USA cities showing that access to urban green space is a function of income and race (i.e., negatively correlated with poverty and being a person of color) [73].

Another problem addressed in the literature was that of social networks as a ‘replacement’ for inefficient markets. Calori [74], using Italian cities as an example, analyzed the operation of ‘micro-networks’—of neighborhood contacts, parishes, associations and informal and family contacts in the provision of food and other essential services and products from the perspective of functioning during a pandemic. McGuirk et al. [75] indicated that the pandemic triggered a resurgence and increase in civic action in the form of mutual aid and ‘pandemic solidarity’. Lombardozzi et al. [76] pointed to the existence of local grassroots social solidarity strategies to counter food poverty during particular pandemic waves. Tricarico [77] emphasized that the social and related health care crisis is not an episodic event but rather a ‘training ground/laboratory’ for place-oriented innovation and proximity ties—advocating for the implementation of the proximity economies framework, which may represent an unexplored context in which to address policies able to tackle the disparities between territories and the inequalities among different social groups.

The next thematic thread that emerged in the articles related to civic participation. Wilkinson [46] emphasized a need for a greater participation by and collaboration between informal leaders of informal urban settlements and local governments in public health and reducing social inequality. Fabris et al. [78] emphasized the role of participation in planning for healthy cities. A second strand pointed to the flourishing of e-participation and increased citizen participation in public discussions by analyzing good practices [42]. 

The analyzed articles also signaled the importance of: social innovation in post-COVID-19 scenarios [79,80]; technology—including the Internet of Things and increasing digitization—in the functioning of cities and their communities (especially in smart cities) and the impact of digitization on public health and urban governance [49,81] and linking social issues to SDG implementations, the European Green Deal and green infrastructure developments in cities [65,79].

### 3.3. Spatial Organization

Issues of spatial organization were raised quite frequently in discussions of health-policy changes (in a total of 66 articles) but without a strong specificity. One of the most frequently discussed issues was envisioned and advocated changes in broad spatial policy with varying degrees of integration with health objectives [3,39,77,82]. There was a strong consensus that it is especially up to urban planners to enhance the capacities of urban areas to combat pandemic situations (not only those related to the SARS-CoV-2 virus).

Integrated spatial planning [31,83] was indicated as an overarching tool (or rather, a systemic direction for the operationalization of various specific tools). Urban resilience was often mentioned in this context [84,85]. However, it was cogently noted that epidemics were known in antiquity, so the problem is universal, and future urban organization should use proven experience from the past [41]. Of course, the experience should be modified given the scale of the COVID-19 pandemic and advances in technology.

However, the desired operationalization of this generally valid goal of a greater integration and coherence of planning did not lead to more comprehensive solutions for local and regional planning in conjunction with health policy. Frequent demands for changes in spatial organization often descended only to the architectural level, emphasizing the role of the local designs of buildings and public spaces between them (i.e., shaping closed and semi-open spaces) [86]. An extreme form was purely engineering demands (e.g., demonstrating the positive roles of triangular, diagonal and generally ‘curved’ geometric figures in the design of so-called urban furniture to reduce the deposition of cough droplets) [47].

Quite often, the proposed concepts revolved around well-known solutions, such as reducing social contact through, for example, changes in mobility and transport [87] as well as ‘geohygiene’: decontaminating public places or improving aero-sanitary conditions [31,47,88]. The latter highlighted long-established ways to ‘green’ urbanized spaces in the forms of greater proportions of green and blue infrastructures [89] as ‘they have innumerable economic, social, climate and health benefits in addition to their positive impact on pandemic mitigation’ [90]. A similar argument was made for ideas of even broader implementations of changes in spatial mobility (i.e., promoting ‘anti-epidemic’ forms of transport, including bicycles and bike lanes) [78,91] and generally individualizing transport. Such solutions, however, have some inherent contradictions as after all, undoubtedly epidemiologically dangerous public transport has long been considered ‘green’.

The exceptions included work in which, among many other issues, the problem of optimizing and adapting land use to the needs of consumption (land consumption) was recognized [92]. The latter is worth mentioning because it is a quite unique example of a network of cooperation among urban planning students from 19 university centers in Europe, created ‘in the heat’ in the summer of 2020 (i.e., just after the so-called first wave of the pandemic subsided).

Similarly, with exceptions, important issues, such as the re-organization of the network of locations of health care facilities in order to optimize accessibility to these services [26], were not more widely addressed, and if they were, these issues were described incidentally and generally [36,86,93]. Rather, the ‘general’ importance of equal and equitable accessibility to various types of urban goods and services was noted [92]. Nor were examples found anywhere concerning the reorganization of production and distribution in the pharmaceutical industry (i.e., a need to shorten logistics and production chains to territorially smaller areas to ensure production security (in this case, in drug manufacturing)). In this context, in terms of food security, it is worth noting a rather unique work on self-organization and social networks in supplying oneself with food and drugs in Italy [75]. In contrast, there was quite a lot of work generally showing the benefits of so-called urban agriculture in the times of the pandemic and lockdowns [94,95].

The question of post-pandemic urban space was inextricably linked to futuristic and, as it turned out, increasingly close visions of a post-pandemic future formulated in the past. Key issues, such as digitization, automation and robotization, were highlighted [96,97], but it was difficult to find papers or more profiled pieces of work explicitly addressing the more in-depth issue of health care organization in the future.

### 3.4. Governance and Tasks of Authorities in Cities

Issues related to re-defining the tasks of public authorities were included in 41 publications. The dominant portion of them (37 publications) referred to issues of strengthening the roles of public health in development policy. Ding et al. [35] recognized the negative impacts of urbanization on the spread of the virus. A corollary of the above even called for curbing urbanization-related trends, including limiting human interactions, until an effective cure for COVID-19 is found [98]. The dilemmas associated with the above, including the human rights crisis, pose, according to Dockerill et al. [83], challenges for cities to ensure resilience (which include reducing populations’ vulnerabilities to infection). It is important to highlight the identification of this postulate as a task of urban public authorities. Wang and Mao [99], analyzing public health strategies in select countries during pandemic waves, identified diverse practices. These included building and re-building hospitals as well as providing new beds for patients.

Some authors in particular emphasized the global synchronization of health services as valuable. The World Health Organization’s [39] position was that cities must have plans for managing health services during a pandemic. A demand to go beyond health care structures in public policies also applied to the European Union [100]. The development of social care was indicated as a necessary element (a lack in this area leads to negative consequences). Chornyi [101] postulated a transformation of the health care system connected to the inclusion of private funds as part of its financing (resulting from a deeper cooperation between the state and business). Another direction connected to the greater inclusion of business was broadening innovations in urban management—also in the dimension including public health [75].

The pandemic-induced re-centralization and decentralization of public authorities were also discussed in the literature. Glinka [102] pointed out that the capacity of state authorities is incomparably greater than the insufficient capacity of local (municipal—including urban) authorities. Clemens [100] pointed out that the role of the European Union should be strengthened in this context. It is not about the simple transfer of competencies but rather institutional innovations. He proposed the (1) EU support of crisis coordination and collection of comparative data; (2) the coordination of the mutual support of individual countries, (3) the inclusion of public health in the EU surveillance process, (4) the coordination of inter-country stocks of countermeasures and (5) the coordination of cooperation with the WHO.

However, there were also voices emphasizing the roles of local authorities. With respect to Germany, a good cooperation with central authorities, taking into account the perspectives of subnational authorities, contributed to pandemic containment [103]. De Biase [104] argued for decentralization, pointing out that decentralized pandemic responses lead to a greater heterogeneity in response patterns within a country. 

## 4. Discussion

Based on the literature review that was conducted, it is important to point out that issues related to the broad problem of urban health care were broadly reflected in the individual studies reviewed. To the widest extent, this applied to the health and e-social sphere. Issues concerning the organization of space and management directly connected with health care occurred in a more limited scope.

In the realm of the various issues related to direct changes in the health sector during the COVID-19 pandemic, the following discussion threads were identified:

- Pandemic risks create a need to balance the challenges of maintaining the health and quality of life of urban residents with the challenges of the economic costs incurred to do so.
- Pandemic risks require coordinating and combining the efforts of various professionals at different administrative and territorial levels to create a strong health care system—‘a people-centered primary health care approach, and resilient systems, societies and communities’ [39].

A study by Primc and Slabe-Erker [105] showed that the most important challenge in making decisions on health risk prevention is to maintain a balance between the reduction of the risk of a SARS-CoV-2 transmission and economic costs, taking into account the principles of sustainable development. The issue of striking a balance between health objectives and economic costs thus remains a key challenge, embedded in the discussion of balancing rationales, in shaping public interest [106]. The variability and diversity of a health situation (and its risks) caused by a pandemic lead to the conclusion that this balancing must be continuous, dynamic and based on flexible criteria. It also requires the coordination of disparate services and an interdisciplinary approach. Other long-term implications are also important. Megahed and Ghoneim [36] pointed to a need for sustained reorganization of health care services even after the pandemic is over. As Hoernke [60] pointed out, each post-pandemic city will require a unique recovery strategy. The rapid, unsustainable urbanization occurring in cities around the world in the face of a pandemic crisis will likely increase the demand for basic urban services, such as housing and health care.

All this confirmed the problem of the increasing the influence of health determinants and health care on diverse spheres. The pandemic, on the one hand, allowed for a much better recognition of these challenges and, on the other hand, began a redefinition of urban policies. The COVID-19 pandemic demonstrated a ‘necessity to put health as a high priority in public policies and expenditure and to prepare cities to upcoming risks and external events’ [42]. To this end, it seems important to combine the efforts of public authorities, policy specialists, practitioners and research theorists. As it turned out, the COVID-19 pandemic exposed inequalities in the sphere of social and economic relations, including health inequalities, but at the same time provided a unique opportunity to provide ‘a faster, more coordinated and coherent global response for strengthening cities’ resilience and sustainability’. While there is no universal pathway for further urban development in the post-COVID-19 era, the urban crisis caused by COVID-19 has created an opportunity to develop universal synthetic solutions to enhance the safety and quality of life of urban residents.

The literature highlighted a need for post-pandemic strategies for cities, which should lead to reduced social inequalities and more participatory governance, including urban planning (e.g., in the contexts of public spaces, publicly accessible internet and sanitation densities correlated with population density). It also highlighted that the redress of social inequalities should be combined with responsible housing policies by cities and approaches to building more resilient cities [31,60].

Rocco et al. [59] emphasized that the pandemic has been recognized as an opportunity to bring about far-reaching transformations of societies (especially congested urban areas) toward sustainability and equity. The literature mainly discussed this, while in reality, there have been few signs of systemic change. Actions for public spaces, the creation of green spaces and new bike paths were emphasized, but there was little talk of addressing the structural causes of inequality. The discourse in the literature was more concerned with the diagnosis of social inequalities translating into health inequalities and their impacts on the severity of the pandemic with little attempt to focus on necessary future actions. The need for the participation of stakeholders, including excluded marginalized groups (e.g., residents of informal settlements and representatives of various minorities) in building healthier post-pandemic cities was emphasized. As Wray et al. [62] highlighted, the pandemic revealed in many cities a lack of space in the public realm for people, and consequently, many urban health inequalities and insecurities will be exacerbated during future pandemics, heat waves and climate-related migrations.

The directions of change in the discussion of urban spatial organization were not entirely clear. On the one hand, it is almost certain that there will have to be an architectural reconstruction (and, for objective reasons, an urban reconstruction, to a lesser extent) as there are overlapping pre-pandemic trends related to digitization (smart cities), greening (green cities) and, finally, energy and climate challenges (economical/efficiency cities). On the other hand, the pro-health concept of the city is not entirely clear as it is not yet mature and detailed. In this context, Bandarin et al. [107] pointed out that as a result of pandemic and post-pandemic trends, we are likely to face an ‘expanded’ city that may be more equitable or more repressive depending on the prevailing local politics.

In general, the weak prominence of the issue of urban-space organization in the discussion of the health drivers of development policy may be due to two reasons. The first reason is that there are still many uncertainties related to the functioning of urban systems, including overarching issues, such as the role of public spaces in a pandemic world [108] and the responsibilities of different groups (‘actors’) in society. The second reason is pragmatic and stems from the fact that it is difficult to imagine a more radical reconstruction of urban layouts, especially of large cities, as this requires huge organizational and financial outlays, including raw materials (well-known in this context are concepts of building medium-sized cities from ‘raw roots’ (e.g., in Arab countries and South Africa, estimated at $100 billion per city). Without such a reconstruction, it is difficult to create bold visions of a health city.

Thus, the discourse focused on what seems feasible under realistic organizational, logistical and financial (and perhaps political) conditions (i.e., revitalizing existing building layouts and other infrastructures and focusing on architectures and logistics (including transportation) or reducing urban planning to the scale of a neighborhood or a few adjacent streets). While in the first moment after the outbreak of the pandemic in Europe, slogans about the radical reconstruction of cities (and, by extension, the whole world) were frequent [109] and especially uttered by prominent and thus ‘loud’ authors [110], with the passing of time, pragmatism won out, or perhaps a kind of cooling and habituation took place.

Finally, unnoticed were the technological developments ‘standing around the corner’ that may radically change urban mobility (e.g., individual drones that move automatically like cars). The COVID-19 pandemic can and should accelerate the spread of such solutions, just as in this context the collapse of the Iron Curtain in the late 1980s proved to be a breakthrough for the development of IT and mobile telephony, resulting in the ‘liberation’ of technologies until then reserved only for the military.

In the sphere of issues involving the role of public authorities in relation to the pandemic, two lines of discussion could be identified in the scope of the analysis:

- A broader integration of public health into public policies [99].
- Changes in the powers of public authorities at different levels [102,103,104].

The former can be considered both in the context of an ad hoc response to successive waves of a pandemic and in the context of developing a long-term pattern of action by public authorities. In the case of an immediate response, the exchange of experience between countries is crucial. In developing long-term responses, it is important to emphasize that urbanization, unfortunately, contributes to an increase in infections, even with specialized health services [35]. This, moreover, distinguishes urban areas from rural areas. It is in cities that the need to adapt public policies seems particularly important. This makes integrated development planning all the more important, as a result of which the health component should be part of spatial policy, tax policy, property management, social policy and environmental policy. The health component, based on detailed analyses, may even be the main basis for such a development policy integration. Regardless of the above, it is worth looking for solutions related to public comanagement. It is public comanagement that constitutes an opportunity and a point of reference that should be taken advantage of, if only within the framework of the argument to include private entities in the process of financing health care [101].

Public comanagement is also noticeable when discussing the re-formulation of public authorities in relation to pandemics. Regardless of their point of view, authors emphasized the necessity of such comanagement [103]. At the same time, assuming that city authorities are unable to fight a pandemic on their own, their roles in defining current challenges and needs seem to be very important. Such definitions should be done within the framework of the developed model of integrated development planning. The wider involvement of the European Union and other international organizations in these processes [100] should also be considered as an interesting postulate.

The authors are aware of some limitations with their research. The detailed processing and classification of a large number of publications requires some time. In the meantime, there are, of course, further publications that could also provide an important reference point. Therefore, this article should be regarded as a statement in the discussion, which, however, requires further development. Furthermore, it should be noted that the article focuses on academic publications, which often differ in practice between specific countries and cities. The latter sphere also deserves analyses, which should be based on detailed case studies. Nevertheless, scholarly analyses can be very helpful in making syntheses. The diversity of political, social and spatial systems in different countries means that the alignment of the two spheres (scientific and practical) should be done very carefully. Certain limitations also result from the contents of some of the analyzed publications. Very often, these publications were formulated at certain levels of generality. Some authors did not concretize more directional guidelines. This was often understandable; sometimes, it is difficult to draw more general conclusions from analyses of specific case studies. Approaches of a more comprehensive nature are lacking. Nevertheless, such attempts at future publications should be made to a much greater extent. This applies especially to issues of spatial organization, which are very important from the analyzed perspective. Moreover, it should be noted that in determining the directions of changes, the authors sometimes too easily referred to the inclusion of given issues within the development strategy. This was of course necessary, but the indicated postulate appeared on many other occasions and was insufficient. Therefore, in subsequent analyses, it will be worth trying, at least directionally, to develop more detailed models in this respect (perhaps referring to types of countries and types of cities). The approach to health challenges itself also needs strengthening. In the existing literature (which is fully understandable), these challenges were too often presented in undefined ways. Sometimes, they were framed as ad hoc responses to current threats, and in other cases, as broader concepts of more general (long-term) changes. This direction should also be developed.

## 5. Conclusions

The 240 publications analyzed (mainly articles that appeared in academic journals) allowed us to assess the discussion of the relationship between health and urban policies (their inter-relationships and inter-dependencies). In the analysis, we did not refer to the characteristics of individual cities, their categories or their sizes. We were interested in urban policy postulates regardless of which cities they might apply to. This issue is interesting and will require further consideration and research, especially at the stage of operationalizing new postulated concepts. First, and this is the most general conclusion, the postulates identified in the four groups were very much inter-linked, from which it can be concluded that the discussion is not yet advanced (i.e., more specialized and ‘nuanced’).

As for more specific findings, they are as follows. The health perspective identified a need for a balance between health care and economic costs and a need to coordinate diverse professionals/spheres of action. This is linked to demands regarding governance (modifications of the competencies of public authorities) and social aspects (the reduction of social inequalities).

However, there is still a great deal of uncertainty about how the broader linkage of health issues and urban policy should look, especially whether it requires re-defining social and spatial approaches. As pointed out in the analysis, post-pandemic cities should be more socially cohesive, and tackling social inequalities is a strategy for building more resilient communities. It seems, however, that research on how to effectively address the social inequalities that have built up during the pandemic has not yet reached the realm of implementation.

In the above context, the findings of the available studies suggested that an urban health-oriented research approach should be adopted in which urban strategies and project activities aim to continuously improve people’s health. This was particularly emphasized by Fior and Mpampatsikos [111] and Bandarin et al. [106], who called for more research on cultural, legal and political differences in pandemic control effectiveness. It would be interesting in the future to relate these studies to the levels of social and health inequalities that were observed. In the undertaken research, it will be important to refer to categories of cities (e.g., shrinking cities, historic cities or dynamic growth centers) and their sizes (small, medium, large or metropolis).

The relationship between public and individual health and the organization of urban space is particularly complex and multi-faceted. In conclusion, however, it must be acknowledged that the entirely valid postulate of ‘designing cities for health’ [35] still needs a theoretical concretization and a lot of effort to be put into practice. In this context, further lines of analysis can be suggested. These include each of the issues identified in the article. It seems crucial to work out a balance (and on the scale of municipal policy) between health care and economic costs. Such a balance also requires social and spatial analyses, illustrating the variations in the social situations of individual cities (and more broadly in urban areas, including, sometimes, vast suburbs) and its relationship (both in terms of causes and consequences) to the intensities of pandemics and other health threats. Such research may, in part, contribute to clarifying the call for ‘health urban design’ and re-defining (both at the city level itself and at other levels of government influencing urban policy) the remit of individual public authorities.

## Figures and Tables

**Table 1:** Number of publications obtained based on keyword combinations.

	1st Word	Cities	City	Town	Urban	Spatial Policy	Spatial Planning	Public Health	Social Exclusion	Social Inclusion	Health Education	Total
2nd Word												
COVID		804	915	163	1455	4	3	1992	14	27	366	5743
SARS-CoV-2		29	215	7	82	0	0	247	0	2	6	588
pandemic		280	240	29	646	2	1	969	6	10	171	2354
epidemic		37	121	11	85	0	0	206	0	0	15	475
coronavirus		157	355	33	114	1	1	448	0	0	41	1150
Total		1307	1846	243	2382	7	5	3862	20	39	599	10,310

Source: Own elaboration based on Google Scholar/Publish or Perish.

**Table 2:** Publications related to thematic groups.

General Issue	Specific Issue	Number of Articles
Direct demands for changes in health care	Reorganization of the health care system	17
	Increase in the role of prevention of social and civilization diseases in cities	4
	Activities to maintain urban physical and emotional wellbeing (physical and emotional wellbeing)	9
	Promoting healthy lifestyles to city dwellers	13
	Monitoring the health statuses of urban residents	10
	Urban health resources and operations	6
	Urban health funding	4
Social issues	Social determinants of health	34
	Reducing or addressing health disparities	20
	Special measures for the most vulnerable demographic groups of a population (elderly, families with 2, 3 or more children, single mothers or fathers raising children and young people not studying or working)	10
	Specific actions for the most vulnerable population groups (people with disabilities and chronic illnesses, people with low socio-economic statuses (SESs), people struggling with addictions, etc.)	17
	Urban health education	9
Spatial organization	Changes in spatial policy (integration with health objectives)	39
	Reorganization of the distribution of health care facilities	8
	Reorganization of the distribution of social care facilities	2
	Promotion of ‘anti-epidemic’ forms of transport (bicycles, bicycle paths and other infrastructures to disperse movement)	27
	Local supply chain assurances (e.g., the concept of feeding zones) in the pharmaceutical industry	11
	Improvement of aero-sanitary conditions (e.g., city ventilation)	11
Redefinition of the tasks of public authorities in the face of indicated challenges	Decentralization (increases in the roles of local governments in health care management)	8
	Recentralization (increases in the roles of states in managing health care)	6
	Strengthening health issues in development policy	37

Source: Own elaboration.

## Data Availability

Not applicable.

## Abbreviations

PHSM	Public health and social measures
DPTT	Digital proximity tracing technology
DDEIS	Data-driven epidemic intelligence strategies
GDP	Gross domestic product
NGOs	Non-governmental organizations
SDGs	Sustainable development goals
